# Zenker’s Diverticulum Presenting With Complete Esophageal Obstruction in a 55-Year-Old Male

**DOI:** 10.7759/cureus.90241

**Published:** 2025-08-16

**Authors:** Omar R Zayed, Urmimala Chaudhuri, Hassan Zreik, Drew J Triplett

**Affiliations:** 1 Boonshoft School of Medicine, Wright State University, Dayton, USA; 2 Internal Medicine Residency Program, Wright State University, Dayton, USA; 3 Gastroenterology, Wright State University Boonshoft School of Medicine, Dayton, USA

**Keywords:** airway disorders, cricopharyngeal myotomy, dysphagia, esophageal obstruction, swallowing disorder, transcervical diverticulectomy, zenker’s diverticulum

## Abstract

Zenker’s diverticulum is an outpouching of mucosa through Killian’s dehiscence secondary to increased intraluminal pressure. It typically affects older adults, particularly males over the age of 60. Common symptoms include dysphagia, halitosis, regurgitation, and chronic cough. We present an atypical case of Zenker’s diverticulum in a younger patient, who presented with shortness of breath and was found to have near-complete esophageal obstruction. A 55-year-old male with a history of hypertension presented to the emergency room with progressive shortness of breath. Gastroenterology was consulted for several months of dysphagia to both solids and liquids, accompanied by a 25 lb unintentional weight loss. He denied prior food bolus impaction. Initial evaluation included a video fluoroscopic swallow study with recommendations of a liquid diet. An esophagram revealed a large Zenker’s diverticulum causing near-complete obstruction of the upper thoracic esophagus. Esophagogastroduodenoscopy (EGD) demonstrated severe upper esophageal stricture with complete obstruction and retained food material. A computed tomography (CT) scan of the soft tissue neck confirmed a large Zenker’s diverticulum with significant compression of the proximal esophagus. Otolaryngology was consulted, and the patient underwent an esophagoscopy with transcervical Zenker’s diverticulectomy. Postoperative esophagram showed no evidence of a leak. His diet was gradually advanced, and he was discharged in stable condition. This case highlights an uncommon presentation of Zenker’s diverticulum, including complete esophageal obstruction and respiratory symptoms in a younger patient. Early identification and a multidisciplinary team were critical for successful outcomes.

## Introduction

Zenker’s diverticulum (ZD) is an acquired outpouching of the mucosa and submucosa through a zone of relative weakness in the posterior hypopharyngeal wall known as Killian’s triangle [1]. ZD is a relatively rare disorder, with a reported prevalence of approximately 0.01-0.011% in the general population [2]. A large national cohort study estimated an annual incidence of 2.9 per 100,000 person-years. ZD primarily affects older adults, with a median age at diagnosis of 72 years, and occurs more commonly in men [3].

The typical presenting symptoms of ZD are progressive dysphagia and regurgitation of undigested food [4]. Other common features include halitosis from food retention, chronic cough, and unintended weight loss [5-7]. We report an atypical case of ZD in a relatively younger patient who presented with dyspnea and was found to have near-complete esophageal obstruction. This case emphasizes the importance of early recognition, appropriate imaging, and multidisciplinary management.

## Case presentation

A 55-year-old Caucasian male with a history of hypertension presented to the emergency department with progressive shortness of breath, fatigue, and palpitations. He was found to be in atrial fibrillation with rapid ventricular response and was diagnosed with acute hypoxemic respiratory failure secondary to influenza pneumonia with suspected bacterial superinfection.

On initial examination, the patient was ill-appearing but alert and oriented. He was tachycardic with dry mucous membranes, a clear oropharynx, and normal peripheral perfusion. Lung auscultation revealed clear breath sounds without wheezes or crackles. The abdomen was soft, non-tender, and non-distended. Key laboratory findings on presentation are summarized in Table 1. His hospital course was complicated by persistent hypoxemia requiring BiPAP and high-flow nasal cannula support, acute kidney injury, metabolic encephalopathy, and paroxysmal atrial fibrillation requiring anticoagulation and rate control.

During the initial days of admission, nursing staff noted the patient was having difficulty swallowing medications. Further investigation revealed that the patient had several months of progressive dysphagia. He reported difficulty swallowing solids, specifically pasta and meat, which later progressed to difficulty swallowing liquids. Additionally, he noted frequently needing to slowly chew food into small pieces and use fluids to help food pass. He endorsed coughing up undigested food and a sensation of food getting stuck in the lower throat. He denied any history of food impaction and aspiration but reported an unintentional weight loss of approximately 25 pounds over the previous month. 

**Table 1:** Key Laboratory Findings on Presentation BUN: blood urea nitrogen; BUN/Cr: blood urea nitrogen to creatinine ratio; AST: aspartate aminotransferase; ALT: alanine aminotransferase; ALP: alkaline phosphatase; WBC: white blood cell count

Lab Finding	Result	Reference Range
Sodium (mmol/L)	135	135-145
Potassium (mmol/L)	3.5	3.5-5.0
Chloride (mmol/L)	97	98-107
Carbon dioxide (mmol/L)	18	22-29
Anion gap (mmol/L)	20	8-16
BUN (mg/dL)	120	7-20
Creatinine (mg/dL)	4.6	0.6-1.3
BUN/Cr Ratio	28	10-20
AST (U/L)	35	10-40
ALT (U/L)	36	7-56
ALP (U/L)	58	40-129
Total bilirubin	0.7	0.1-1.2
Albumin (g/dL)	3.6	3.5-5.0
Troponin T (ng/L)	25	<14
WBC (white blood cells × 10^9^/L)	8.7	4.0-11.0
Hemoglobin (g/dL)	14.5	13.5-17.5
Platelet count (× 10^9^/L)	180	150-450

**Figure 1:**
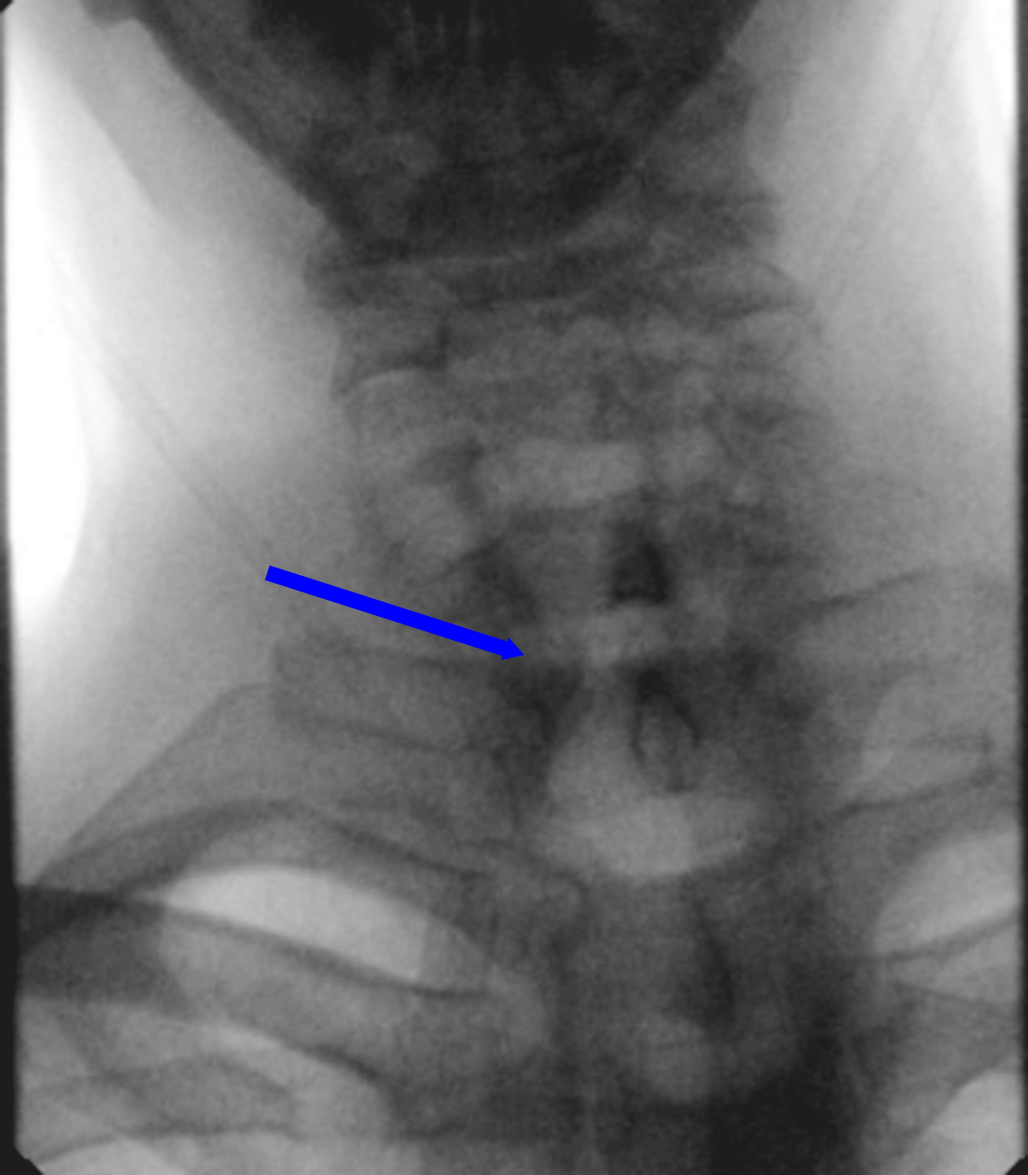
Barium esophagram showing a large Zenker’s diverticulum causing near obstruction of the upper thoracic esophagus.

**Figure 2:**
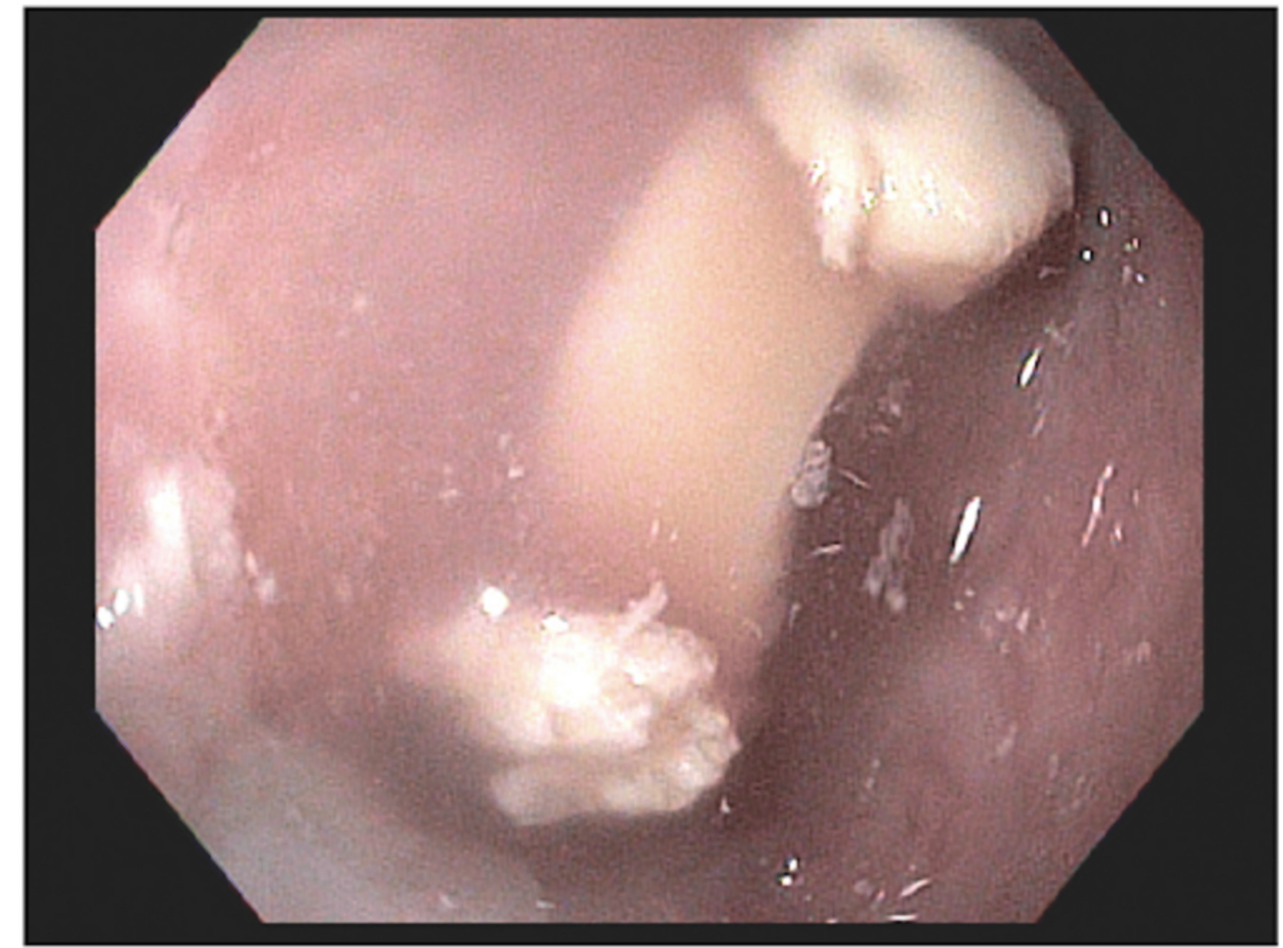
EGD highlighting severe stricture with retained food contents. EGD: Esophagogastroduodenoscopy

**Figure 3:**
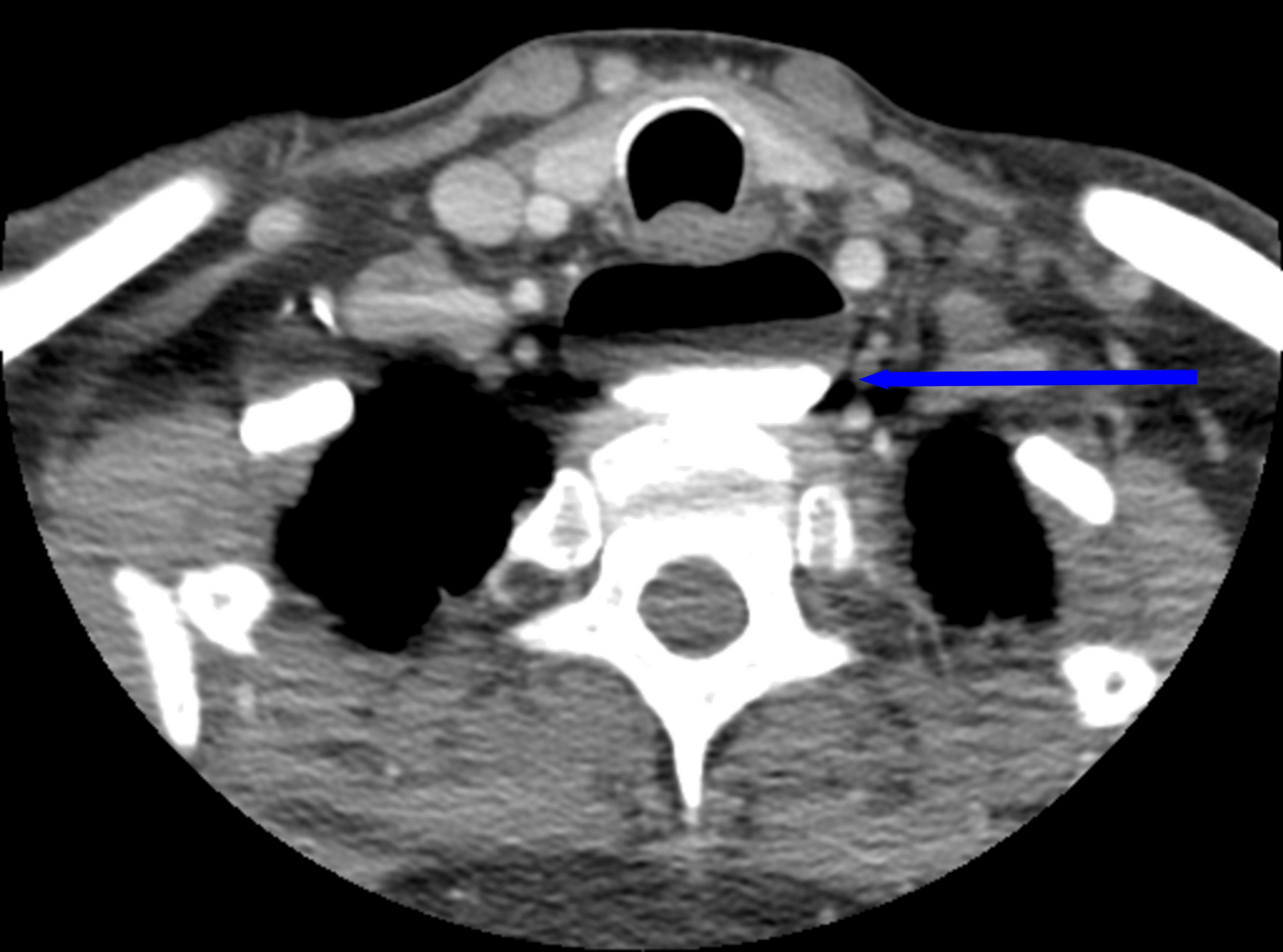
CT soft tissue neck showing a large Zenker’s diverticulum with compression of the proximal esophagus.

Once the patient was medically stable, a fiberoptic endoscopic evaluation of swallowing was performed by speech pathology, which revealed trace aspiration with thin liquids and spillage from the piriform sinuses. Additionally, decreased hyolaryngeal elevation and reduced tongue base retraction were noted. Subsequently, the patient was placed on a full liquid diet. Gastroenterology was then consulted for further evaluation. A barium swallow revealed a large ZD causing near-complete obstruction of the upper thoracic esophagus (Figure 1). Gastroenterology proceeded with an esophagogastroduodenoscopy (EGD), which revealed a severe stricture (Figure 2). The scope could not be advanced beyond the stricture, and retained food debris was noted proximal to the site. Biopsies were taken for histologic evaluation and revealed unremarkable squamous esophageal epithelium. A subsequent computed tomography (CT) scan of the neck and chest confirmed a markedly enlarged ZD measuring approximately 5.7 × 4.7 × 2.6 cm, causing anterior compression of the proximal esophagus and occupying substantial space in the cervical region (Figure 3).

Given the findings and the severity of obstruction, otolaryngology was consulted for definitive management. The patient underwent an operative endoscopic exam under general anesthesia followed by a transcervical Zenker’s diverticulectomy with cricopharyngeal myotomy. Intraoperatively, a large diverticulum was identified and successfully resected via a left neck approach, and the cricopharyngeal muscle was divided to relieve the underlying outflow obstruction.

A postoperative contrast esophagram showed intact esophageal continuity and no evidence of a leak. Postoperatively, the patient’s diet was gradually advanced from liquids to soft solids. Additionally, the patient demonstrated resolution of his dysphagia and respiratory symptoms. He was discharged in stable condition.

## Discussion

ZD is an acquired pulsion diverticulum of the hypopharynx, characterized by herniation of the mucosa and submucosa through Killian’s triangle [1]. Killian’s triangle is located between the oblique fibers of the thyropharyngeus and the horizontal fibers of the cricopharyngeus muscle. The underlying pathophysiology is multifactorial, but a primary component seems to be dysfunction of the cricopharyngeal muscle. This dysfunction leads to incomplete relaxation and decreased compliance of the cricopharyngeus during swallowing, resulting in increased hypopharyngeal pressure. Over time, the elevated pressure causes herniation of the pharyngeal mucosa through Killian’s triangle [8-10]. It is classically a disorder of older adults, typically presenting in the seventh to ninth decades of life. Risk factors include advanced age, male sex, and possibly conditions associated with cricopharyngeal muscle dysfunction [3]. Additionally, there is an association with gastroesophageal reflux disease, which may contribute to the pathogenesis by increasing the resting tone of the cricopharyngeus [11]. Possible complications of large diverticula include chronic weight loss, aspiration pneumonia, and rarely upper airway compression [12].

In our case, the patient was only 55 years old, which is uncommon and clinically significant. Additionally, the patient lacked the typical risk factors associated with ZD, making the etiology of its development at his age unclear. Further distinguishing this case is the severity of the patient’s presentation. Dysphagia and regurgitation of undigested food are the hallmark symptoms in the majority of patients. Patients often report halitosis, a sensation of food getting stuck in the throat, and a chronic cough after eating [1]. Strikingly, our patient’s initial presentation was marked by dyspnea and acute respiratory failure from pneumonia, with his significant dysphagia only recognized during the hospital course. His markedly enlarged diverticulum produced mechanical airway compression, likely exacerbating the pneumonia and prompting airway interventions throughout his hospitalization. While he later endorsed months of dysphagia, regurgitation, and substantial weight loss, he had tolerated or overlooked these symptoms until decompensation occurred.

The diagnosis of ZD is based on a combination of clinical suspicion and confirmatory imaging. Possible differential diagnoses include esophageal malignancy, esophageal stricture, motility disorders, Schatzki ring, and cricopharyngeal achalasia. The criterion standard for diagnosis is a barium swallow study, which can provide information on the size and morphology of the diverticulum [13,14]. Once ZD is diagnosed, endoscopic examination is used to exclude malignancy [15]. Other imaging modalities, such as CT and EGD, may play a role in diagnosis and treatment planning.

However, EGD should only be used as an adjunct to barium swallow, as it has limitations: the diverticulum can be missed, and there is a risk of perforation when advancing the endoscope [16]. Our patient initially underwent fiberoptic endoscopic evaluation of swallowing due to concerns for dysphagia, which raised suspicion for structural pathology. This prompted a barium swallow study, which confirmed a large ZD (5.7 cm), followed by EGD and CT imaging to further assess the extent of obstruction, rule out malignancy, and guide surgical planning.

The main treatment modalities for ZD include open surgical approaches (diverticulectomy, diverticulopexy, cricopharyngeal myotomy), rigid endoscopic techniques, flexible endoscopic techniques, and Zenker per-oral endoscopic myotomy (Z-POEM). Endoscopic approaches have become the preferred therapy for most patients, as comparative outcomes have shown that endoscopic approaches are associated with shorter hospital stays, lower perioperative morbidity, and faster recovery compared to open surgery. Although endoscopic techniques can be used to treat large ZDs, open surgical approaches are used in patients with unfavorable anatomy that may not be amenable to endoscopic intervention. In our patient, the diverticulum measured over 5 cm and caused near-complete esophageal obstruction with airway compression. Given the size, severity of symptoms, and failure to traverse the stricture endoscopically, an open transcervical diverticulectomy with cricopharyngeal myotomy was successfully performed [17-19]. Future research should focus on prospective comparative studies evaluating long-term outcomes, recurrence rates, and complication profiles of emerging endoscopic techniques versus traditional open approaches. Additionally, research into the pathophysiology of ZD, such as genetic, anatomical, and motility factors, may reveal more about the mechanisms of disease progression and recurrence.

## Conclusions

This case highlights an atypical presentation of ZD in a relatively young patient with severe respiratory compromise and near-complete esophageal obstruction. Despite months of progressive dysphagia and weight loss, the underlying pathology went undiagnosed until an acute decompensation prompted a multidisciplinary evaluation. Prompt recognition of dysphagia and appropriate imaging led to the diagnosis. Given the diverticulum’s size and failed endoscopic passage, an open transcervical diverticulectomy with cricopharyngeal myotomy was performed. This case highlights the potential for ZD to cause mechanical airway compromise and esophageal obstruction, which are atypical presenting symptoms. Early identification and multidisciplinary management remain essential for favorable outcomes.
